# Endothelial Function, Inflammation, Thrombosis, and Basal Ganglia Perivascular Spaces in Patients with Stroke

**DOI:** 10.1016/j.jstrokecerebrovasdis.2016.08.007

**Published:** 2016-12

**Authors:** Xin Wang, Francesca M. Chappell, Maria Valdes Hernandez, Gordon Lowe, Ann Rumley, Kirsten Shuler, Fergus Doubal, Joanna M. Wardlaw

**Affiliations:** * Division of Neuroimaging Sciences, Centre for Clinical Brain Sciences, Edinburgh, UK; † Institute of Cardiovascular and Medical Sciences, Royal Infirmary, University of Glasgow, Glasgow, UK; ‡ NHS Lothian, Edinburgh, UK

**Keywords:** Endothelial function, stroke, small vessel disease, perivascular spaces

## Abstract

**Background and Objective:**

Recent studies suggest perivascular spaces are a marker of small vessel disease, blood–brain barrier permeability, and inflammation, but little is known about their risk factors and associations with peripheral blood markers.

**Materials and Methods:**

In prospectively recruited patients with recent minor ischemic stroke, we investigated the influence of age, sex, hypertension, diabetes, and smoking on the severity of perivascular spaces in the basal ganglia seen on T2-weighted magnetic resonance imaging. We assessed plasma markers of endothelial function (von Willebrand factor, intracellular adhesion molecule-1), inflammation (interleukin-6, tumor necrosis factor-alpha, C-reactive protein), and thrombosis (fibrinogen, prothrombin fragments 1 + 2, thrombin–antithrombin complex, tissue plasminogen activator, D-dimer). We used a validated semi-automated method to measure basal ganglia perivascular spaces count and volume. We tested uni- and multivariable associations between blood markers and basal ganglia perivascular spaces count and volume.

**Findings:**

In 100 patients (median age: 67 years, range: 37-92), on adjusted analysis, basal ganglia perivascular spaces count was associated with age (r = .117, *P* = .003) and hypertension (r = 2.225, *P* = .013). On multivariable linear regression, adjusted for age, sex, hypertension, smoking and diabetes, reduced von Willebrand factor was associated with increased basal ganglia perivascular spaces count (r = −.025, *P* = .032).

**Conclusion:**

The association of increased basal ganglia perivascular spaces count with reduced von Willebrand factor is novel. As von Willebrand factor may promote cerebral endothelial integrity, insufficient von Willebrand factor is consistent with dysfunctional cerebral endothelium and increased basal ganglia perivascular spaces in cerebral small vessel disease. Quantitative perivascular spaces measurement may increase sensitivity to detect cerebral endothelial dysfunction.

## Introduction

Perivascular spaces (PVS), or Virchow–Robin spaces, are pial extensions of the subarachnoid space that surround the arteries, arterioles, veins, and venules as they penetrate the brain parenchyma.^1^, ^2^ PVS are an important drainage conduit for soluble and insoluble material through the central nervous system.^3^ Many inflammatory processes take place in PVS; for example, PVS are a specific site for immune cell accumulation, reaction, and transmigration into the brain parenchyma (e.g., leukocytes, dendritic cells, T-cells, B-cells, and macrophages^4^, ^5^, ^6^).

PVS on magnetic resonance imaging (MRI) are an imaging marker for cerebral small vessel disease (SVD).^7^, ^8^, ^9^ PVS are associated with other SVD features, such as white matter hyperintensities (WMH), atrophy, microbleeds, lacunes, and recent small subcortical infarcts.^10^ Increasing numbers of PVS were also associated with increased blood–brain barrier (BBB) permeability in stroke patients,^11^ worse cognitive function in older people,^12^ and more WMH in older subjects^13^ and in patients with stroke.^7^, ^8^

Blood marker levels in the peripheral circulation could reflect endothelial function, inflammation, and thrombosis changes in the brain, and are associated with SVD features such as WMH and lacunar stroke.^14^ However, only four studies explored the associations between blood markers and PVS.^13^, ^15^, ^16^ More PVS in the basal ganglia (BG) regions were associated with higher plasma oxidized low-density lipoprotein in adjusted analysis, suggesting that oxidized low-density lipoprotein may contribute to PVS progression through endothelial dysfunction and antibody formation.^16^ Associations between more BG PVS and higher plasma neopterin (from activated monocytes or macrophages),^15^ plasma interleukin-6 (IL-6),^17^ and plasma C-reactive protein^13^ suggested associations with inflammation in patients with stroke,^15^, ^16^ vascular disease,^17^ and older community-dwelling individuals,^13^ respectively.

These studies used visual assessment of PVS that reflects the number of visible PVS, but PVS may increase in size as well as in number, and the visual score may lack sensitivity in detecting associations with plasma markers. We developed a computational quantitative method to measure PVS volume and number.^18^ As it is unclear if PVS reflect primarily inflammation, endothelial dysfunction, thrombosis, or all three, in the present analysis we test associations between PVS count and volume and plasma markers of endothelial function, inflammation, and thrombosis in the chronic phase after lacunar stroke or mild cortical stroke.

## Methods

### Patient Recruitment and Assessment

We used data from 100 patients recruited prospectively with lacunar or minor (i.e., non-disabling) cortical ischemic stroke who participated in a study of BBB permeability.^19^ All patients were carefully examined by an experienced stroke physician who recorded the full medical history and risk factors, including hypertension, diabetes, smoking, heart disease, and cholesterol. All patients had diagnostic brain MRI, including sagittal T1-weighted and axial diffusion-weighted imaging, T2-weighted, fluid-attenuated inversion recovery, and gradient-echo sequences acquired on a GE Signa scanner (General Electric Medical Systems, Milwaukee, Wisconsin, USA) at 1.5T, to classify final stroke subtype and qualitatively assess PVS (sequence details published previously^7^, ^19^). All patients received guideline-based stroke secondary prevention (clopidogrel, statin in all, and antihypertensive drugs in patients with hypertension). All patients gave written informed consent to participate in the study and the study was approved by the local ethics committee.

### MRI Data Analysis

All image processing was performed blind to clinical and blood marker details. We used a semi-automated method to measure BG PVS count and volume.^18^ This method uses intensity-normalized structural T2-weighted MRI obtained after performing a linear intensity adjustment with a gamma correction factor of two, followed by linear mapping to the original images, in MATLAB (http://www.mathworks.co.uk/help/images/ref/imadjust.html). We, then, manually applied a standard region-of-interest extraction, limiting the assessment of PVS to two bilateral ovoid regions on a representative BG slice, one in each hemisphere, delineated by the vertical ramus of the lateral fissures and the posterior segment of the lateral fissures, and automatically extracted PVS using the Analyze 10.0 software (AnalyzeDirect, Inc., Overland Park, Kansas, USA). This method showed a strong relationship with visual scores, with regression coefficients of 2.114 (95% confidence interval: 1.364-2.864, *P* < .001) for BG PVS count and .022 (95% confidence interval: .012-.031, *P* < .001) for BG PVS volume.

### Blood Marker Assessment

We collected venous blood from each patient approximately 2 months (minimum of 1 and maximum of 3 months) after stroke to avoid the acute stroke phase. Blood samples were put immediately into two 2.5-mL ethylenediaminetetraacetic acid tubes and an 8-mL tube containing clot activator and gel. The samples were transferred from the ward on water ice, centrifuged at 2000 g for 10 minutes, and stored at −80°C until analysis.

Blood marker analysis was performed in an accredited laboratory, blinded to the patients' clinical and imaging details. We measured 10 blood markers using high sensitive assays (assay details in Supplementary Table S1):

1) endothelial dysfunction: von Willebrand factor (vWF) and intracellular adhesion molecule-1;
2) inflammation: IL-6, tumor necrosis factor-alpha, and C-reactive protein; and
3) thrombosis: fibrinogen (Fib), prothrombin fragments 1 and 2 (F 1 + 2), thrombin–antithrombin complex (TAT), tissue plasminogen activator (tPA), and D-dimer.

### Statistical Analysis

Blood marker data were incomplete for 7 of the 100 patients, but all available data were used in the analyses.

We investigated the influence of patients' demographics (age, sex, hypertension, smoking, and diabetes) on (1) PVS severity (count and volume) and (2) blood marker levels using multivariable linear regression. We then assessed the associations between PVS severity (count and volume) and blood marker levels using univariable and multivariable linear regression (adjusted for patients' demographics). To investigate smoking further, we condensed the four original categories (non-smoker; recent smoker, less than a year; long-term light smoker; heavy smoker) into “never smoked” or “current or ever smoked” and used this dichotomized smoking variable in most of our analysis. Smoking status was missing for one patient.

All analyses were performed using Minitab (Minitab Inc, version 16) (Minitab Inc., State College, Pennsylvania, USA). Alpha level for significance was *P* < .05.

## Results

Of the 100 patients, 51 had lacunar and 49 had cortical ischemic stroke. The mean age was 69 years old, ranging from 37 to 92 years old. The median National Institute for Health Stroke Scale score was 2; 62% of patients had hypertension, 53% had smoking history, and 15% had diabetes. The median time to blood sampling was 54.4 days (Q1-Q3: 36-74).

### BG PVS Count and Volume and Patient Demographics

In the 100 patients, the median of PVS count was 10, and the median of PVS volume was .09 mL. Univariate linear regression (Table 1) showed that both BG PVS count (*P* = .003) and volume (*P* = .020) increased with age and BG PVS count (*P* = .013) increased in hypertension, although the association between hypertension and BG PVS volume did not reach significance. No associations were seen between BG PVS count and volume and gender, smoking, or diabetes on univariate analysis.

### Blood Markers and Patient Demographics

On multivariable analysis, the endothelial function marker vWF was associated with age (*P* < .001). The inflammatory marker IL-6 (*P* = .048), and the thrombosis markers tPA (*P* = .001) and Fib (*P* = .016), were associated with smoking. No significant associations were seen between any blood markers and sex, hypertension, or diabetes (see Supplementary Table S2).

### Blood Markers, BG PVS Levels, and Risk Factors

Univariable analysis showed associations between BG PVS count and thrombosis markers F 1 + 2 (*P* = .050) and TAT (*P* = .013), and between BG PVS volume and TAT (*P* = .037), but not with any other blood markers (Supplementary Table S3). After adjusting for age, sex, and risk factors, the associations between TAT, F 1 + 2, and BG PVS became non-significant, but the blood endothelial function marker vWF became negatively associated with BG PVS count (*P* = .032) (Table 2, Table 3).

## Discussion

We found that PVS increased with age and hypertension on multivariable analysis, but not with smoking or diabetes. We also found that most of the blood markers that we studied were not significantly associated with PVS count and volume, but that there was a negative association between BG PVS count and the endothelial function marker vWF after adjustment for age and risk factors. This association was the opposite to what would be expected if this were simply due to increasing age or smoking, and supports the concept that endothelial dysfunction is a contributing factor in the pathogenesis of cerebral SVD.

High vWF level is considered a sign of endothelial cell activity. vWF is increased in the plasma of patients who subsequently develop stroke,^20^ and with a variety of neurologic conditions such as cerebral malaria^21^ and severe head injury.^22^ The potential protective effect of vWF on BBB permeability was demonstrated recently in a vWF knockout mouse model: In normal mice, vWF inhibited the expression of the tight junction protein claudin-5 and led to increased BBB permeability, but in the vWF knockout mice increased claudin-5 expression alone did not protect the BBB but was detrimental, suggesting that vWF may be important for the regulation of cerebral endothelial function and BBB permeability during certain disease states.^23^ Lack of vWF expression could, therefore, indicate impaired cerebral endothelial integrity and increased BBB permeability, consistent with our findings using gadolinium-enhanced brain MRI in the same population.^19^ Further examination of the role of vWF is needed as it may influence BBB function through effects on tight junction proteins, which are impaired in an SVD model^24^, ^25^ and are thought to be impaired in human SVD.

Smoking affects some blood markers. In multivariable analysis, the inflammatory blood marker IL-6 and thrombosis blood markers Fib and tPA were significantly associated with smoking. However, we did not find significant associations between PVS and smoking status, consistent with the 3C-Dijon study of 1818 patients, 108 (<10%) of whom were current smokers,^9^ whereas in our study there was a much higher proportion (53%) who smoked. We were unable to explain the effect of smoking on blood markers by the 4-year age difference between smoker and non-smoker. A larger sample is needed to explore the associations between PVS and smoking.

We drew blood approximately 2 months after onset of minor stroke, so our results reflect the chronic and not acute phase after stroke, and we are neither able to comment on blood markers and time after stroke, nor can we comment on blood markers and antiplatelet or other secondary prevention drugs, as all patients were prescribed routine secondary prevention agents. The associations between increasing age, hypertension, and BG PVS count and volume may not have been completely corrected statistically and may confound the association of PVS and vWF, both of which normally rise with age. However, associations with age and hypertension are consistent with previous studies showing that PVS severity increases with age and hypertension.^6^, ^8^, ^9^, ^26^, ^27^, ^28^

The study strengths include the careful blinding of all analyses and the validated PVS and blood marker quantification methods, and the range of biomarkers assessed representing three important domains of vascular function. These PVS associations need to be confirmed in larger samples, accounting for other factors such as structural brain volume and SVD markers, demographic and vascular risk factors, and perhaps at a wider range of times after stroke. They should also examine if relationships differ for PVS count and PVS volume, as the two features may provide different information about the role of PVS in the pathogenesis of brain disease.

## Figures and Tables

**Table 1:** Associations between BG PVS count and volume and patient demographics (age, gender, hypertension, smoking, and diabetes, univariable linear regression)

Coefficient (95%CI, *P*)	BG PVS count	BG PVS volume
Age	.117 (.040 to .194, *P* = .003*)	.001 (.000 to .002, *P* = .020*)
Male sex	1.433 (−.518 to 3.383, *P* = .148)	.023 (−.002 to .047, *P* = .072)
Hypertension	2.225 (.476 to 3.973, *P* = .013*)	.022 (−.001 to .044, *P* = .056)
Smoking	−.648 (−2.315 to 1.019, *P *= .442)	−.009 (−.030 to .012, *P *= .383)
Diabetes	−.756 (−3.086 to 1.574, *P *= .521)	−.001 (−.031 to .028, *P *= .920)

Abbreviations: BG, basal ganglia; CI, confidence interval; PVS, perivascular spaces.

BG PVS count: total number of PVS dots in each patient in the selected BG regions; PVS volume: total volume of all the PVS dots; age: age increase per year; smoking: current or ever smoked versus never smoked.

* *P* < .05.

**Table 2:** Associations between BG PVS count and blood markers after adjusting for patient demographics (age, sex, hypertension, smoking, and diabetes)

Blood marker	Blood marker association	Age	Male sex	Hypertension	Smoking	Diabetes
Endothelial function						
vWF (n = 98)	−.025	.155	1.364	2.095	−.498	−.576
	(−.047, −.002)	(.072 to .239)	(−.605 to 3.333)	(.365 to 3.824)	(−2.164 to 1.168)	(−2.949 to 1.797)
	*P* = .032*	*P* > .001*	*P* = .172	*P* = .018*	*P* = .554	*P* = .631
ICAM-1 (n = 95)	−.003	.109	1.259	2.408	−.849	−.440
	(−.019 to .013)	(.031 to .187)	(−.798 to 3.316)	(.617 to 4.199)	(−2.563 to .866)	(−2.950 to 2.070)
	*P* = .747	*P* = .007*	*P* = .227	*P* = .009*	*P* = .328	*P* = .728
Inflammation						
IL-6 (n = 97)	−.153	.123	1.377	2.158	−.532	−.697
	(−.549 to .242)	(.044 to .201)	(−.646 to 3.401)	(.374 to 3.942)	(−2.279 to 1.215)	(−3.135 to 1.742)
	*P* = .443	*P* = .003*	*P* = .180	*P* = .018*	*P* = .547	*P* = .572
TNF-α (n = 98)	−.613	.114	1.290	2.259	−.654	−.710
	(−1.650 to .424)	(.037 to .192)	(−.724 to 3.304)	(.502 to 4.016)	(−2.346 to 1.037)	(−3.121 to 1.701)
	*P* = .243	*P* = .004*	*P* = .206	*P* = .012*	*P* = .444	*P* = .560
CRP (n = 98)	−.034	.118	1.473	2.304	−.525	−.801
	(−.127 to .060)	(.041 to .196)	(−.548 to 3.494)	(.528 to 4.081)	(−2.251 to 1.201)	(−3.234 to 1.631)
	*P* = .474	*P* = .003*	*P* = .151	*P* = .012*	*P* = .547	*P* = .514
Thrombosis						
Fib (n = 96)	−.639	.123	1.371	2.091	−.335	−.649
	(−2.175 to .896)	(.044 to .201)	(−.668 to 3.409)	(.309 to 3.872)	(−2.102 to 1.431)	(−3.087 to 1.788)
	*P* = .410	*P* = .003*	*P* = .185	*P* = .022*	*P* = .707	*P* = .598
F 1 + 2 (n = 98)	.002	.111	1.413	2.165	−.507	−.568
	(−.002 to .006)	(.032 to .190)	(−.598 to 3.425)	(.395 to 3.935)	(−2.234 to 1.220)	(−3.019 to 1.883)
	*P* = .424	*P* = .006*	*P* = .166	*P* = .017*	*P* = .561	*P* = .646
TAT (n = 98)	.122	.109	1.379	2.069	−.426	−.661
	(−.020 to .264)	(.032 to .186)	(−.609 to 3.366)	(.318 to 3.820)	(−2.120 to 1.269)	(−3.054 to 1.731)
	*P* = .091	*P* = .006*	*P* = .172	*P* = .021*	*P* = .619	*P* = .584
tPA (n = 98)	−.074	.116	1.495	2.267	−.475	−.726
	(−.351 to .204)	(.039 to .194)	(−.547 to 3.536)	(.495 to 4.040)	(−2.279 to 1.330)	(−3.152 to 1.700)
	*P* = .600	*P* = .004*	*P* = .149	*P* = .013*	*P* = .603	*P* = .554
D-dimer (n = 98)	.001	.116	1.382	2.209	−.634	−.663
	(−.003 to .005)	(.038 to .194)	(−.638 to 3.402)	(.440 to 3.979)	(−2.337 to 1.068)	(−3.105 to 1.779)
	*P* = .661	*P* = .004*	*P* = .178	*P* = .015*	*P* = .461	*P* = .591

Abbreviations: BG, basal ganglia; CRP, C-reactive protein; Fib, fibrinogen; ICAM-1, intracellular adhesion molecule-1; IL-6, interleukin-6; PVS, perivascular spaces; TAT, thrombin–antithrombin complex; TNF-α, tumor necrosis factor-alpha; tPA, tissue plasminogen activator; vWF, von Willebrand factor.

PVS count: total number of PVS dots in each patient; age: age increase per year; smoking: current or ever smoked.

* *P* < .05.

**Table 3:** Associations between BG PVS volume and blood markers adjusted for patient demographics (age, sex, hypertension, smoking, and diabetes)

Blood marker	Blood marker association	Age	Male sex	Hypertension	Smoking	Diabetes
Endothelial function						
vWF (n = 98)	1.28E-04	.001	.022	.021	−.008	3.03E-04
	(4.19E-04 to 1.62E-04)	(.000 to .002)	(−.004 to .047)	(−.001 to .043)	(−.030 to .013)	(−.030 to .031)
	*P* = .383	*P* = .014*	*P* = .095	*P* = .065	*P* = .453	*P* = .984
ICAM-1 (n = 95)	0.53E-04	.001	.019	.024	−.009	.004
	(−1.51E-04 to 2.57E-04)	(.000 to .002)	(−.007 to .046)	(.001 to .047)	(−.031 to .013)	(−.028 to .036)
	*P* = .606	*P* = .026*	*P* = .145	*P* = .038*	*P* = .400	*P* = .804
Inflammation						
IL-6 (n = 97)	−2.27E-04	.001	.020	.019	−.010	.002
	(−.005 to .005)	(.000 to .002)	(−.005 to .045)	(−.003 to .041)	(−.032 to .011)	(−.029 to .032)
	*P* = .927	*P* = .011*	*P* = .113	*P* = .096	*P* = .351	*P* = .908
TNF-α (n = 98)	−.002	.001	.021	.022	−.009	−4.18E-04
	(−.015 to .011)	(.000 to .002)	(−.004 to .047)	(−.001 to .044)	(−.030 to .013)	(−.031 to .030)
	*P* = .757	*P* = .022*	*P* = .100	*P* = .056	*P* = .412	*P* = .978
CRP (n = 98)	−2.59E-04	.001	.022	.022	−.008	−.001
	(−.001 to .001)	(.000 to .002)	(−.003 to .048)	(.000 to .045)	(−.030 to .014)	(−.032 to .030)
	*P* = .664	*P* = .020*	*P* = .087	*P* = .053	*P* = .467	*P* = .945
Thrombosis						
Fib (n = 96)	−.001	.001	.021	.021	−.007	.001
	(−.021 to .018)	(.000 to .002)	(−.005 to .047)	(−.002 to .043)	(−.030 to .015)	(−.031 to .032)
	*P* = .906	*P* = .024*	*P* = .116	*P* = .075	*P* = .515	*P* = .971
F 1 + 2 (n = 98)	0.16E-04	.001	.022	.021	−.008	.001
	(−0.35E-04 to 0.67E-04)	(.000 to .002)	(−.004 to .047)	(−.001 to .043)	(−.029 to .014)	(−.030 to .032)
	*P* = .535	*P* = .031*	*P* = .091	*P* = .065	*P* = .490	*P* = .946
TAT (n = 98)	.001	.001	.021	.020	−.007	1.84E-04
	(−.001 to .003)	(.000 to .002)	(−.004 to .047)	(−.002 to .042)	(−.028 to .015)	(−.030 to .031)
	*P* = .163	*P* = .031*	*P* = .094	*P* = .078	*P* = .538	*P* = .990
tPA (n = 98)	−.001	.001	.023	.022	−.006	−.001
	(−.005 to .002)	(.000 to .002)	(−.002 to .049)	(.000 to .045)	(−.029 to .017)	(−.031 to .030)
	*P* = .451	*P* = .022*	*P* = .075	*P* = .050*	*P* = .604	*P* = .974
D-dimer (n = 98)	0.16E-04	.001	.021	.021	−.009	.001
	(−0.39E-04 to 0.71E-04)	(.000 to .00)	(−.004 to .047)	(−.001 to .044)	(−.030 to .013)	(−.030 to .031)
	*P* = .571	*P* = .023*	*P* = .099	*P* = .061	*P* = .414	*P* = .973

Abbreviations: BG, basal ganglia; CRP, C-reactive protein; Fib, fibrinogen; ICAM-1, intracellular adhesion molecule-1; IL-6, interleukin-6; PVS, perivascular spaces; TAT, thrombin–antithrombin complex; TNF-α, tumor necrosis factor-alpha; tPA, tissue plasminogen activator; vWF, von Willebrand factor.

PVS count: total number of PVS dots in each patient; age: age increase per year; smoking: current or ever smoked.

* *P* < .05.
