# Supplementary material for: Endothelial Function, Inflammation, Thrombosis, and Basal Ganglia Perivascular Spaces in Patients with Stroke
Source: J Stroke Cerebrovasc Dis. 2016 Dec;25(12):2925–31. doi: 10.1016/j.jstrokecerebrovasdis.2016.08.007 (PMC5176093; doi:10.1016/j.jstrokecerebrovasdis.2016.08.007)
Supplement: Appendix S1 — Tables S1-S3. [file mmc1.docx]

**Supplementary Table 1.** Assay details for blood markers.

| **Blood markers** | **Measurement method** | **Unit** | **Intra-assay CV** | **Inter-assay CV** |
| --- | --- | --- | --- | --- |
| Endothelial function |  |  |  |  |
| vWF | ELISA (DAKO, High Wycombe, UK) | IU/dL | 3.3% | 4.2% |
| ICAM-1 | ELISA (R&D Systems, Abingdon, UK) | ng/ml | 3.6% | 7.4% |
| Inflammation |  |  |  |  |
| IL-6 | ELISA (R&D Systems, Abingdon, UK) | pg/ml | 7.5% | 8.9% |
| TNF-á | ELISA (R&D Systems, Abingdon, UK) | pg/ml | 8.4% | 12.5% |
| CRP | Immunonephelometry  (Prospec, Dade Behring Milton Keynes, UK) | mg/l | 4.7% | 8.3% |
| Thrombosis |  |  |  |  |
| Fib | Immunonephelometry  (Prospec, Dade Behring Milton Keynes, UK) | g/l | 7.5% | 8.9% |
| F 1+2 | Immunonephelometry  (Prospec, Dade Behring Milton Keynes, UK) | pg/ml | 6.0% | 9.0% |
| TAT | Immunonephelometry  (Prospec, Dade Behring Milton Keynes, UK) | ng/ml | 3.6% | 4.3% |
| tPA | ELISA (Biopool AB, Umea Sweden) | ng/mL | 6.6% | 6.5% |
| D dimer | ELISA (Biopool AB, Umea Sweden) | ng/ml | 4.7% | 5.2% |

ELISA: enzyme-linked immunosorbent assay, CV: coefficients of variation, vWF: von-Willebrand factor, ICAM-1 intracellular adhesion molecule 1; IL-6interleukin-6, TNF-α: tumor necrosis factor alpha, CRP: C-reactive protein; Fib: Fibrinogen, F 1+2: prothrombin fragments 1 and 2, TAT: thrombin-antithrombin complex, tPA: tissue plasminogen activator.

Supplementary Table 2. Univariable associations between blood markers and patient demographics (age, gender, hypertension, smoking, and diabetes, univariable linear regression).

| **Blood markers** | **Age** | **Male Sex** | **Hypertension** | **Smoking** | **Diabetes** |
| --- | --- | --- | --- | --- | --- |
| Endothelial function |  |  |  |  |  |
| vWF (n=98) | 1.565  (0.870 to 2.260) p<0.001* | -1.815  (-19.897 to 16.266),  p=0.842 | -5.428  (-21.275 to 10.419), p=0.498 | 5.499  (-9.764 to 20.762), p=0.476 | 5.966  (-15.794 to 27.727), p=0.587 |
| ICAM-1 (n=95) | -0.487  (-1.510 to 0.537) p=0.347 | -0.758  (-27.848 to 26.331), p=0.956 | -13.698  (-37.106 to 9.710), p=0.248 | -7.535  (-30.061 to 14.991), p=0.508 | -5.699  (-38.728 to 27.330), p=0.733 |
| Inflammation |  |  |  |  |  |
| IL-6 (n=97) | 0.021  (-0.020 to 0.062) p=0.306 | 0.044  (-1.022 to 1.110), p=0.935 | -0.015  (-0.955 to 0.925), p=0.974 | 0.910  (0.010 to 1.811), p=0.048 | -0.179  (-1.463 to 1.105), p=0.782 |
| TNF-á (n=98) | -0.004  (-0.019 to 0.012) p=0.621 | -0.193  (-0.593 to 0.207), p=0.339 | 0.049  (-0.301 to 0.400), p=0.781 | -0.034  (-0.371 to 0.304), p=0.844 | 0.022  (-0.460 to 0.503), p=0.929 |
| CRP (n=98) | 0.036  (-0.135 to 0.208)  p=0.675 | 1.900  (-2.567 to 6.366), p=0.400 | 2.240  (-1.674 to 6.154), p=0.259 | 3.228  (-0.542 to 6.998), p=0.092 | -2.317  (-7.692 to 3.058), p=0.394 |
| Thrombosis |  |  |  |  |  |
| Fib (n=96) | 0.008  (-0.003 to 0.018), p=0.161 | 0.178  (-0.097 to 0.454), p=0.202 | 0.013  (-0.230 to 0.256), p=0.913 | 0.287  (0.054 to 0.520), p=0.016 | -0.126  (-0.457 to 0.205), p=0.452 |
| F 1+2 (n=98) | 3.635  (-0.307 to 7.577), p=0.070 | -2.884  (-105.447 to 99.679) p=0.956 | 38.677  (-51.211 to 128.565), p=0.395 | -77.362  (-163.940 to 9.216), p=0.079 | -94.426  (-217.858 to 29.006), p=0.132 |
| TAT (n=98) | 0.064  (-0.048 to 0.176), p=0.258 | 0.245  (-2.658 to 3.149), p=0.867 | 1.309  (-1.236 to 3.854), p=0.310 | -1.705  (-4.156 to 0.746), p=0.170 | -0.507  (-4.002 to 2.988), p=0.774 |
| tPA (n=98) | -0.005  (-0.063 to 0.052), p=0.852 | 1.170  (-0.332 to 2.673, p=0.125) | 0.527  (-0.790 to 1.843), p=0.429 | 2.165  (0.897 to 3.433), p=0.001 | -0.036  (-1.844 to 1.772), p=0.969 |
| D dimer (n=98) | 0.855  (-2.821 to 4.531), p=0.645 | 27.421  (-68.217 to 123.059, p=0.570) | 20.053  (-63.765 to 103.871), p=0.636 | 0.546  (-80.186 to 81.278), p=0.989 | -62.350  (-177.447 to 52.747), p=0.285 |

Values are coefficient, 95% confidence interval and p value. Age: age increase per year; Smoking: current or ever versus never smoked.

Supplementary Table 3. Univariable association between individual blood marker and BG PVS count/volume (univariable linear regression).

| **Blood markers** | **BG PVS count** | **BG PVS volume** |
| --- | --- | --- |
| Endothelial function |  |  |
| vWF (n=99) | -0.005 (-0.028 to 0.018, p=0.660) | 3.95E-05 (-23.81E-05 to 31.70E-05, p=0.778) |
| ICAM-1 (n=96) | -0.009 (-0.026 to 0.008, p=0.311) | -1.40E-05 (-22.67E-05 to 19.80E-05, p=0.893) |
| Inflammation |  |  |
| IL-6 (n=98) | -0.108 (-0.529 to 0.313, p=0.612) | -1.10E-05 (-0.005 to 0.005, p=0.997) |
| TNF-á (n=99) | -0.702 (-1.825 to 0.422, p=0.218) | -0.003 (-0.017 to 0.011, p=0.640) |
| CRP (n=99) | -0.009 (-0.108 to 0.090, p=0.852) | -4.00E-05 (-124.94E-05 to 117.00E-05, p=0.948) |
| Thrombosis |  |  |
| Fib (n=97) | -0.160 (-1.732 to 1.411, p=0.840) | 0.003 (-0.016 to 0.022, p=0.753) |
| F 1+2 (n=99) | 0.004 (0.000 to 0.008, p=0.050*) | 3.92E-05 (-1.12E-05 to 9.00E-05, p=0.126) |
| TAT (n=99) | 0.189 (0.041 to 0.336, p=0.013*) | 0.002 (0.000 to 0.004, p=0.037*) |
| tPA (n=99) | -0.065 (-0.343 to 0.214, p=0.647) | -0.001 (-0.004 to 0.002, p=0.540) |
| D dimer (n=99) | 0.002 (-0.003 to 0.007, p=0.386) | 2.60E-05 (3.20E-05 to 8.30E-05, p=0.372) |

Values are coefficient, 95% confidence interval and p value. vWF: von-Willebrand factor, ICAM-1 intracellular adhesion molecule 1; IL-6interleukin-6, TNF-α: tumor necrosis factor alpha, CRP: C-reactive protein; Fib: Fibrinogen, F 1+2: prothrombin fragments 1 and 2, TAT: thrombin-antithrombin complex, tPA: tissue plasminogen activator.
